# Insights into the Issue of Deploying a Private LoRaWAN

**DOI:** 10.3390/s22052042

**Published:** 2022-03-05

**Authors:** Radek Fujdiak, Konstantin Mikhaylov, Jan Pospisil, Ales Povalac, Jiri Misurec

**Affiliations:** 1 Faculty of Electrical Engineering and Communication, Brno University of Technology, Technicka 12, 61600 Brno, Czech Republic; xpospi90@vutbr.cz (J.P.); povalac@vut.cz (A.P.); misurec@vut.cz (J.M.); 2 Centre for Wireless Communications, University of Oulu, Erkki Koiso-Kanttilan Katu 3, 90014 Oulu, Finland; konstantin.mikhaylov@oulu.fi

**Keywords:** IoT, LPWA, LoRaWAN, LoRa, indoor, private, public

## Abstract

The last decade has transformed wireless access technologies and crystallized a new direction for the internet of things (IoT). The modern low-power wide-area network (LPWAN) technologies have been introduced to deliver connectivity for billions of devices while keeping the costs and consumption low, and the range of communication high. While the 5G (fifth generation mobile network) LPWAN-like radio technologies, namely NB-IoT (narrowband internet of things) and LTE-M (long-term evolution machine type communication) are emerging, the long-range wide-area network (LoRaWAN) remains extremely popular. One unique feature of this technology, which distinguishes it from the competitors, is the possibility of supporting both public and private network deployments. In this paper we focus on this aspect and deliver original results comparing the performance of the private and public LoRAWAN deployment options; these results should help understand the LoRaWAN technology and give a clear overview of the advantages and disadvantages of the private versus public approaches. Notably, we carry the comparison along the three dimensions: the communication performance, the security, and the cost analysis. The presented results illustratively demonstrate the differences of the two deployment approaches, and thus can support selection of the most efficient deployment option for a target application.

## 1. Introduction

### 1.1. Internet of Things Connectivity

During the last decade, wireless communication technologies have advanced significantly [1], and it is expected that the number of connected devices will reach 26.4 billion by 2026 [2]. These technologies became the primary activators and essence for the new paradigm of the Internet of Things (IoT) [3], and we are now witnessing the rise of IoT applications [4]. Nowadays, the IoT covers many different areas, i.e., sensor networks, telemetry systems, and remote metering. These applications have very specific requirements, such as [5] long battery life, long communication range, a high number of nodes per base station, and high density of nodes. The conventional technologies were not prepared for new applications, and therefore they did not offer sufficient solutions. A new type of wireless solution has been introduced to fulfill these new and specific needs, known as low-power wide-area network (LPWAN) [6].

At present, there are many different technologies recognized as LPWAN, i.e., long-range wireless-area network (LoRaWAN), SigFox, narrowband IoT (NB-IoT), Weightless, Ingenu, Nwave, Waviot, Wi-Fi HaLow, Tlensa, Amber, and many others [7,8]. Each technology slightly differs from the others, but the main LPWAN parameters, such as long battery life, extended communication range, and relatively low cost, stay the same. However, the SigFox, LoRaWAN, and NB-IoT are the most discussed, with the LoRaWAN being currently the most adopted LPWAN technology for the IoT [9]. The LoRaWAN technology is an open global standard provided by the association of companies known as the LoRaWAN Alliance™ [10]. The LoRaWAN might be deployed as a public or private network, unlike other LPWAN such as SigFox or NB-IoT, which offer only a public variant. This feature offers a new perspective of LPWAN with different applications.

A major change in the IoT connectivity landscape is expected after the introduction and broad deployment of the 5G (fifth generation) mobile networks. The 5G networks will enable much lower latency, higher capacity, and the higher bandwidth compared to 4G technologies [11]. Notably, the 5G [12] will bring the evolution for NB-IoT and LTE-M. However, as discussed in [13], there is no universal solution or a single technology that fits all applications. Each connectivity option is more or less suitable for a specific application. In the same manner, the 5G NR (new radio) technology is of great interest and may enable a whole plethora of new use cases for tactile IoT in the context of industrial and medical verticals, for example. However, the communication range of high-bandwidth 5G connectivity is rather limited, and, as of today, not so many 5G-enabled locations are present in Czech Republic. For this reason, we expect that LPWAN technologies will still continue playing an important role in the future, while the 5G technology can serve their backbone links and more demanding applications and use cases.

### 1.2. Contribution and Structure of This Study

Many works focus on the technology itself or deal with either private or public network independently, as we discuss further in Section 2. The main contribution of this work is that we address both these deployment options and highlight the difference between them, with respect to three major metrics: the network performance, security, and costs. Namely, we first conduct an experimental measurement campaign to estimate the coverage and signal levels of the private and public LoRaWAN networks in the campus of Brno University of Technology, and compare the results of the two networks. Second, we deliver the analysis of the security procedures specified in the different LoRaWAN specification releases, and discuss the security aspects relevant to private and public network deployments. Third, we deliver the model and identify the cost components allowing to estimate the costs of LoRaWAN deployment and owning, and detail the steps one has to take to make a decision whether to go for public or private LoRaWAN deployment. To the best of our knowledge, the current work is the first one which analyzes and compares the two LoRaWAN deployment options, thus supporting selection of the one most suitable.

The rest of the paper is organized as follows: Section 2 provides an in-depth analysis of the advances in LPWAN focusing on the LoRaWAN technology. Section 3 introduces the experimental environment used for our measurements and experiments, while the main contribution (technological, security, and deployment evaluation) is included in Section 4. Finally, Section 5 summarizes our conclusions.

## 2. Background and State of the Art

**Figure 1:**
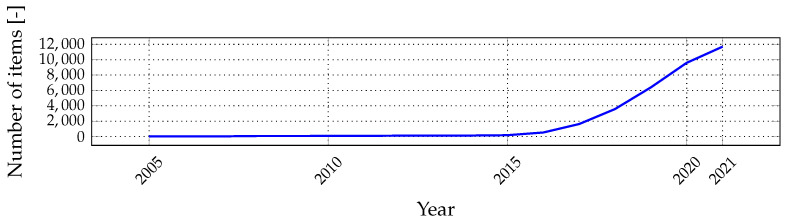
Results of keyword search of the term “LPWAN” in Google Scholar for selected years.

The essence of LPWAN dates back to the 1980s–1990s, when technologies and networks with similar architectures were introduced, i.e., AlarmNet from ADEMCO [14], followed by 2G (second generation mobile network) technologies, and many others. However, the modern technological concept recognized as LPWAN started with SigFox in 2009 and continued with many new technologies such as Weightless, LoRaWAN, Ingenu, Waviot, NB-IoT, and others. Moreover, the first relevant scientific papers about LPWAN were published a few years ago, in 2015; since then, interest in LPWAN has grown steadily (see Figure 1).

LPWAN technologies differ from their precursors as well as from other conventional technologies, i.e., cellular technologies (2G+), mesh technologies (IQRF, Wirepas PINO™), and short-to-middle range radio access technologies (Wi-Fi, ZigBee, Bluetooth, RFID). The main difference is the combination of the long range with a long battery life, which, however, results in the low throughput and limited transmission frequency. This paper focuses on the most widely adopted LPWAN technology, namely LoRaWAN.

**Figure 2:**
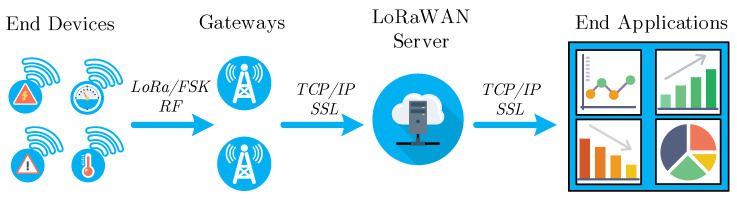
Architecture of the LoRaWAN from the end-device to end-application.

The LoRaWAN is an open standard technology based on the proprietary modulation known as the long-range (LoRa) derivative of the chirp spread spectrum (CSS) modulation. The LoRa modulation was introduced in 2007 by the Cycleo company and was taken over by the Semtech company in 2012. Nowadays, it is protected by patents EP2763321 [15] and US7791415 [16]. On the OSI (open systems interconnection) model layer structure, LoRa can be attributed to the physical layer of LoRaWAN, while LoRaWAN defines the MAC (medium access control) layer. Nowadays, the standard defines two main modulations for terrestrial LoRaWAN (long-range-LoRa; frequency-shift keying—FSK), and the network is deployed in the star topology, similar to cellular networks (see Figure 2). The gateways connect the end-devices (sensors, indicators, meters, and others) via the radio channel, covering selected areas. Subsequently, the data are transmitted via the transport technology (i.e., metallic Ethernet or cellular network) through the LoRaWAN server to the end-user application (e.g., remote monitoring or quality management). The LoRaWAN server is a combination of several different sub-servers—network server (NS), join server (JS), and application server (AS), which are handling different layers and processes (services) [17]. The NS terminates the LoRaWAN MAC layer for end-devices connected to the network. JS manages the over-the-air activation (OTAA) and activation-by-personalization (ABP) processes for end-devices. AS handles all the application layer payloads of the associated end-devices, provides the application-level service to the end-user, and generates all the application layer downlink payloads towards the connected end-devices. However, we will consider the LoRaWAN server as a complex co-located solution for hosting these servers [10].

Many theoretical works and surveys have already been published, focusing on the main parameters of LoRaWAN as well as providing comparison of LoRaWAN and the other LPWAN technologies [9,18,19,20,21,22,23,24]. To give an example, Table 1 illustrates the data rate and the maximum communication range for LoRaWAN as defined by selected papers. The data rate negligibly differs, while the estimated communication range significantly changes through the different papers. The data rate varies mostly because of formal issues, such as not considering the frequency-shift keying modulation or packet overheads of different layers, rounding the values, estimating only the maximal value, and others. On the other hand, the estimates of the maximum communication range differ significantly-from 10 to 50 km; this shows that the experience of various scientists of the LoRaWAN technology differs across the field.

One of the reasons for this is the fact that the main parameters of LoRaWAN strongly depend on many variables, which we discuss below [10,31,32,33,34]:

- **Selected channel (CH) or sub-band (f)** determines the maximum transmit power (10 mW, 25 mW, or 500 mW), which impacts, for example, the communication range, material penetration capability, signal propagation, and the duty-cycle (0.1%, 1%, 10%, or 100%), which impacts the allowed transmission frequency and thus the maximum data rate.
- **Bandwidth (*BW*)** established for Europe is either 125 kHz or 250 kHz.
- **Modulation (MOD)**; LoRaWAN specifies two types of modulation: (i) FSK, and (ii) LoRa modulation. The FSK demands higher signal-to-noise ratio (SNR) and thus is typically used when the communication channel is good and communication range is relatively short. Compared to FSK, the LoRa modulation offers a 13 dB better channel budget and Doppler resistance and approx. 10–20 dB increased interference immunity.
- **Spreading factor (*SF*)** is defined as $SF\in \left\{7;\cdots ;12\right\}$ and determines the symbol duration $T_{s}=2^{SF}T_{c}$, where the chirp interval is defined by *BW* as $T_{c}=1/BW$. Moreover, the *SF* together with *BW* define the physical layer bit-rate:
  (1) $$R_{b}=SF\frac{CR}{\frac{2^{S}F}{BW}}.$$
- **Code rate (*CR*)** is defined as $CR=\frac{4}{4+R}$, where the rate $R\in \left\{0,\cdots ,4\right\}$ and determines redundant bits used for forward error correction—FEC (impacts the ability to correct damaged messages and error-rates). LoRaWAN prescribes use of R=1 for packet payload, and R=4 for the packet header.
- **Device class**, which defines the type of media access for downlink traffic, which also affects the end-device’s power consumption (class A—downlink only after uplink and the minimum consumed power; class B—periodic downlink slots with slightly higher device consumption; class C—highest consumption for devices, but downlink can be sent any time).
- **Device settings** provide a number of other configuration capabilities, including activation (over-the-air activation—OTAA or activation by personalization—ABP), key-generation, firmware updates, data rate (adaptive data rate—ADR, or fixed data rate—FDR), and others).

A lot has been written about the general LoRaWAN parameters. Specifically, a number of the scientific papers provide a general overview of LoRaWAN parameters, metrics, and performance indicators, i.e., capacity [35], coverage [36], maximal range [37], free-space behavior [38], usage [39], energy-efficiency [40], technology comparison [41], performance [42], mobility [43], and other parameters [44]. The authors of [45] compare analytically-obtained parameters of the well-known LPWAN technologies. There are also articles offering datasets from the already functional LoRaWAN network, for the possibility of in-depth research, to mention a few [46,47].

Authors in [19,48] propose routing schemes to create multi-hop communication and routing protocols or decentralized architecture [49] in order to improve LoRaWAN performance. Still, it requires special devices in the network or the end-device modification. The authors in [50] are experimenting with multi-RAT (multiple radio access technology) devices, combining LoRaWAN and NB-IoT to improve mainly energy consumption. The authors of [51] propose LoRaWAN integration into 4G/5G network, where the gateway includes the eNB (LTE evolved Node B) protocols so it can be part of a mobile network. The study [52] examines the technical and economic feasibility of deploying LoRaWAN in a licensed access spectrum band. Currently, however, LoRaWAN network operators use only the unlicensed band.

These results are beneficial for understanding the basics of the LoRaWAN technology or for improving the public network. Nevertheless, the LoRaWAN technology usesboth public and private deployments, and there are major differences between these two approaches (basic differences):

- **Public network**—the network is always owned by a third party (public operator of national or international scale), gateways are deployed to provide coverage over large geographical areas (wide area network—WAN), and for the end-user: fixed parameters of the network, non-transparent and uncontrolled environment, expected lower capital (no need to build the infrastructure), questionable operational expenses (based on the fees and scale), simple and fast deployment, and low technological and management requirements.
- **Private network**—the network is owned by the end-user (i.e., city, company, or individual), gateways are typically deployed to provide coverage over smaller geographical areas (i.e., local, campus, or metropolitan), dynamical (customizable) parameters of network for end-users, transparent and controlled environment for end-users, expected higher capital and questionable operational expenses (based on scale), more complex deployment, and higher technological and management requirements.

Although the private approach is a promising topic, only a very limited number of papers have dealt with private LoRaWAN networks. For example, the authors of [53] work with an experimental self-developed and minimized private network. The paper shows the relation between SNR, data rate, transmission time, and energy consumption. Though the paper provides experimental results, only limited technical details of the experiment are given (i.e., antennas gain and power settings are missing, and information about LoRaWAN stack is missing). Related work [54] focuses on the coverage and signal propagation within the single-gateway network. Authors give sufficient background about the experimental settings and develop a simple visualization method for the chosen use case (apartment building). Another experimental measurement campaign for a private LoRaWAN deployed for industrial application was published in [55]. The paper reports small-scale measurements of signal strength (RSSI) and SNR in an industrial complex (approx. 30 points). Similar work [56] provides results from early-stage measurements of the packet-loss rate in five selected points for a one-floor scenario. The studies [53,54,55,56] report show-cases of early-stage results for the LoRaWAN technology. On the other hand, the work [57] reports complex measurements of power consumption, outdoor signal propagation, adaptive data rate performance, and indoor measurement for a single-gateway network. Notably, the authors control many variables in their measurements, including spreading factor, channel selection, bandwidth selection, and modulation. Another significant work [58] reports very complex results from outdoor measurements of SNR in the campus use case (a single-gateway network). The results from [57,58] are valuable to the scientific community and provide a bright idea about the LoRaWAN technology and its usage in outdoor environment for private use cases. Moreover, the paper [57] presents the approach of using different channels of the 868 MHz band.

When we consider Europe, LoRaWAN operates in the unlicensed sub-GHz band, which for most European countries is set by the standard to 868 MHz [10] under CEPT Rec. 70-03 frequency band regulation [31]. The LoRaWAN specification recommends only three default channels: 868.1 MHz, 868.3 MHz, and 868.5 MHz [10]. In spite of that, they belong to the most frequently used channels in the unlicensed 868 MHz band (863–870 MHz) with a high probability of collision and high level of radio noise. There are several works focusing on collisions in the 868 MHz band, i.e., [9,59,60].

The paper [9] shows decreasing network performance, which comes with the growing number of communicating devices, i.e., decreasing number of received packets, decreasing packet delivery success ratio, and decreasing maximal throughput, to mention only a few. Moreover, the paper [59] shows the growing probability of collisions and packet loss, which comes with the increasing number of communicating nodes operating with different spreading factors. Further, the paper [60] summarizes the relations between the increasing number of communicating devices and the probability of channel occurrence, and the probability of collision. Furthermore, the number of wireless devices is growing in the 868 MHz band every day, which increases the noise background. These are, for example, fire alarm systems, intruder alarm systems, automation systems, access and remote control systems, smart meters, telemetry networks, automotive systems, and many others. The frequency band of 868 MHz is a free-licensed band, and we cannot completely evade the possibility of collision or a higher noise level. The paper [61] summarizes the level of interference experiences for selected channels of the 868 MHz band in different areas: shopping area, business park, hospital complex, industrial area, and residential area. Moreover, the same authors also published the paper [62], which focuses on the impact of interferences on the LoRaWAN and SigFox technologies. The interferences significantly impact the service quality and network coverage. For this reason, the three recommended channels of LoRaWAN will not be sufficient in future.

As one can see from the discussion above, none of the previous works has offered a comparison of the different LoRaWAN deployment options (i.e., private versus public). Meanwhile, this decision is critical and has to be often made by the application developers and service providers. Therefore, to bridge this gap, in the following we discuss the different aspects of the two network deployment options along the three tracks: the communication performance, the security, and the costs. We hope that these results will equip an interested reader with clear understanding of advantages and disadvantages of the two approaches, and assist him or her when deciding whether to go for a private or a public LoRaWAN network.

## 3. Experimental Environment

Our experimental environment is located in Brno, Czech Republic, at the Faculty of Electrical Engineering and Communication Technologies of the Brno University of Technology. The location is covered simultaneously by two LoRaWAN networks—a public and a private one, which we discuss in detail in the following subsections. In the last subsection, we also provide details about the devices we used in our tests.

### 3.1. Private Lorawan

The private network includes one single gateway with LORIOT cloud server. Specifically, we use the Lorank 8+ gateway with the following properties:

- Transmit power up to +27 dBm (500 mW, the power was always based on the selected channel and allowed value from the regulation recommendation [31]).
- Received signal sensitivity up to −138 dBm.
- Five dBi antenna.
- Communication range up to 25 km.
- Up to eight simultaneous receiving channels.
- Up to 60 thousand nodes.
- Whole gateway covered in an IP67 case.

**Figure 3:**
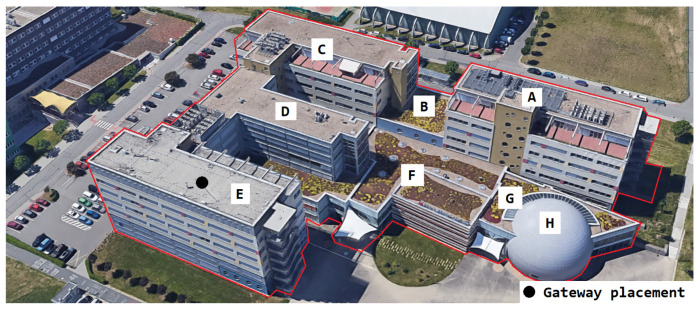
Location of our private one-gateway LoRaWAN experimental network with view of the individual buildings of the Electrical faculty (A–H are the names of the buildings).

**Figure 4:**
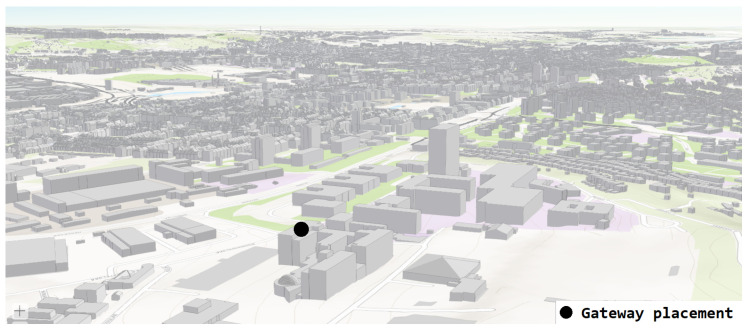
Location of our private one-gateway LoRaWAN experimental network with view of the city of Brno (website: http://webmaps.kambrno.cz/ (accessed on 15 February 2022)).

Figure 3 shows the different parts of the university campus. Each letter indicates one part of the building. The gateway was positioned to cover both the building and part of the city in the E-part of the building. Further, the campus building is one of the highest points in the city. The Lorank 8+ gateway was placed on the roof of the E-part of the building of our campus with the coordinates of the site 49.2269133° N, 16.5752194° E. The E-part of the building provides a power panel for outdoor gateways and metal pillars for deployment. Using the 3D preview of the selected area, it can be observed that from the roof of the E-part of the building, which is marked in the picture (black dot), it is possible to observe most of the buildings in the city of Brno in the line-of-sight (see Figure 4). From the selection of multiple positions, this position seemed the most strategic given the above-mentioned parameters.

**Figure 5:**
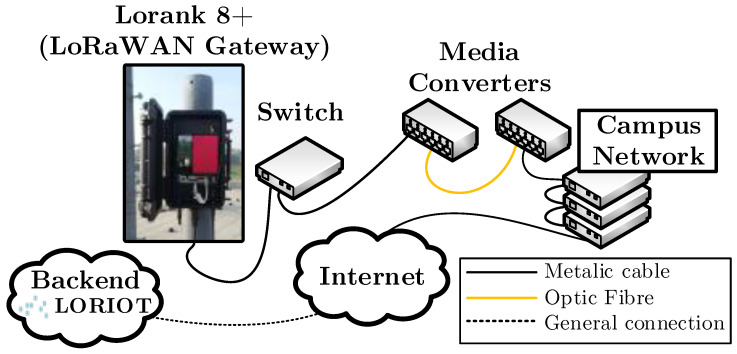
Topology of our LoRaWAN experimental single-gateway network.

The gateway is connected via a basic commercial switch through the 100 MB/s Ethernet to the campus network. The optical connection was chosen to protect network equipment on campus from lightning damage. For this reason, however, Ethernet-to-optic media converter must be used at the gateway side and optic-to-Ethernet media converter on the campus side of the network. The LORIOT cloud itself is not operated locally on campus, but is run directly on LORIOT’s servers, for which gateway access is through the Internet. The complete network topology is displayed in Figure 5.

### 3.2. Public Lorawan

**Figure 6:**
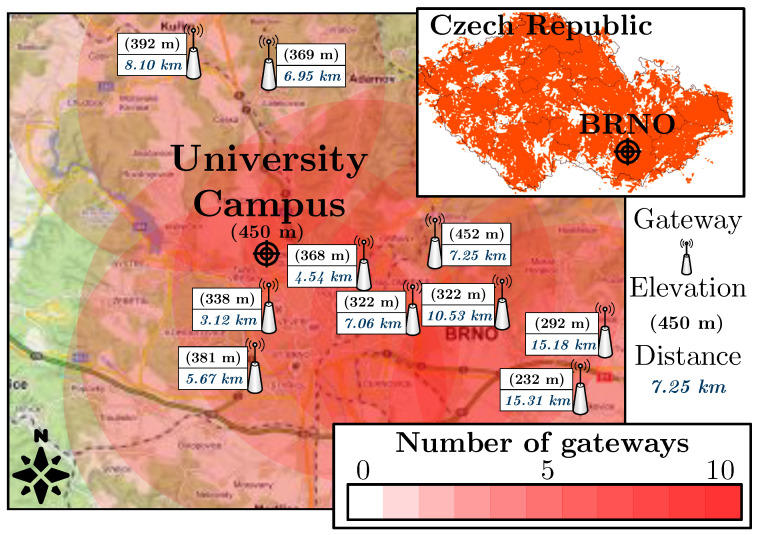
Coverage map of the public LoRaWAN network in the Czech Republic (upper-right corner) and coverage estimation of the nearest public network gateways in the selected location.

To represent the public network, the LoRaWAN network of the Czech national operator ČRa (České Radiokomunikace) was selected (the coverage is displayed in Figure 6). The estimated coverage is based on the theoretical range of 8 km per gateway [58] (the availability was verified in a calibration test in front of the campus building). Therefore, our selected location should be decently covered by the national LoRaWAN operator with multiple nearby gateways. A total of 10 ČRa gateways are located within a radius of 16 km from the university campus. Even though there are several public LoRaWAN providers in the Czech Republic, the selected national operator is the only one covering the whole Czech Republic with hundreds of deployed base stations (WAN area). The other operators mostly provide only local services and their network covers only selected locations (LAN/MAN areas).

### 3.3. Test Device

For our measurements we used a certified LoRaWAN Field Test Device from Adeunis RF (ARF8123AA), with the following parameters [63]:

- Static node (no movement during measurement).
- Transmit power of 14 dBm (25 mW).
- Spreading factor 12.
- ABP activation (OTAA was not supported by public network operator that time), fixed data rate (adaptive data rate was not supported by public network that time).
- Sensitivity up to −140 dBm, 0 dBi wire antenna (Thermolast K TC7AA).
- Communication range up to 15 km.
- Device temperature limits −30 to +70 °C.

To ensure fairness, the frequency plan was chosen based on the public operator’s plan to provide identical conditions for both networks (see Table 2). To minimize the possibility of internetwork collisions, measurements were conducted in different time slots.

## 4. Experimental and Analytical Results

This section contains the main contribution of this paper—the results demonstrating the performance and comparing the two LoRaWAN deployment alternatives along the three tracks (each presents as a separate sub-chapter):

- **Performance evaluation**—provides an evaluation of the performance of the public and the private networks. First, we report the results of the outdoor measurements to confirm our claim about the importance of channel selection and its impact on the network parameters (signal strength, signal-to-noise ratio, and loss rate). Further, we provide extensive experimental measurement results for indoor scenario, coverage, penetration, and loss rate, which should give a sufficient overview of the LoRaWAN behavior in the indoor environment.
- **Security evaluation**—gives accurate information about the recent changes in the LoRaWAN protocol based on the newest documentation. We look at the basics of information security parameters, such as authentication, encryption, and data integrity, but also at key establishment and key update. We also discuss possible vulnerabilities and compare the older with the newest version of the LoRaWAN protocol. Finally, we summarize the differences regarding security in private and public networks.
- **Deployment ease evaluation**—introduces the deployment difficulties, a methodology for the deployment of the public or private network, and evaluates the possible expenses in the context of private and public networks.

### 4.1. Performance Evaluation

#### 4.1.1. Impact of Channel Selection on Network Performance

We measured in front of the building H (approx. 35 m in front of the building H and 60 m from the building E). We selected two frequency plans (see Table 2): (i) the default LoRaWAN channel frequency plan (i.e., the three default LoRaWAN channels in the 868 MHz sub-band); and (ii) the extended channel frequency plan (in the 867 MHz sub-band, including additional channels). The results are displayed in Table 3 (LR = loss rate).

Each value represents an arithmetic mean of 100 messages, which were transmitted over the day for each scenario for the public and private network. The default channel settings showed a higher loss rate (40% higher in the public and 20% higher in the private network). The noise level, when using the default channels, was >14 dB higher than that for the extended frequency plan (the RSSI difference was >26 dB). The measurement sufficiently proves our claim that it is possible to at least partially evade interferences by choosing the right channels to extend the frequency plan. The growing number of devices will, in the future, create an environment with increased noise. Therefore, the LoRaWAN standard will need to evolve together with extending the recommended frequency plan, and give a methodology for choosing the right channels. Therefore, the private network has an advantage (given that these are updated regularly) over the public network as there might be a fully customized frequency plan that allows minimizing the interferences with other systems. Notably, the smaller scale of these networks supports using the different frequency plans in different regions.

#### 4.1.2. Indoor Coverage and Signal Propagation

We measured the public and private network performance in the campus building (see Figure 3). Specifically, we estimated the RSSI, which provides information about signal propagation, coverage, sensitivity, and attenuation of materials. Each value is an arithmetic mean of 20 measurements. These values were obtained for each building part and the floor. Together, they were used to create a heat map of the RSSI (the outdoor calibration values are in Table 3—scenario (ii), private −84 dBm and public −108 dBm).

**Figure 7:**
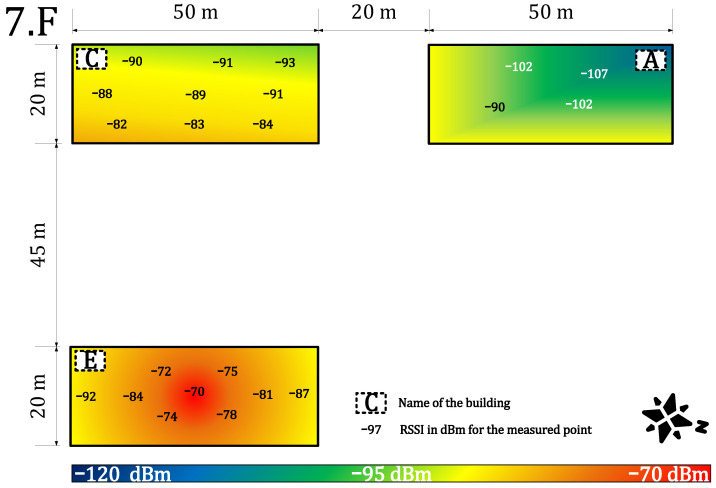
Private network coverage and signal propagation on the seventh floor.

**Figure 8:**
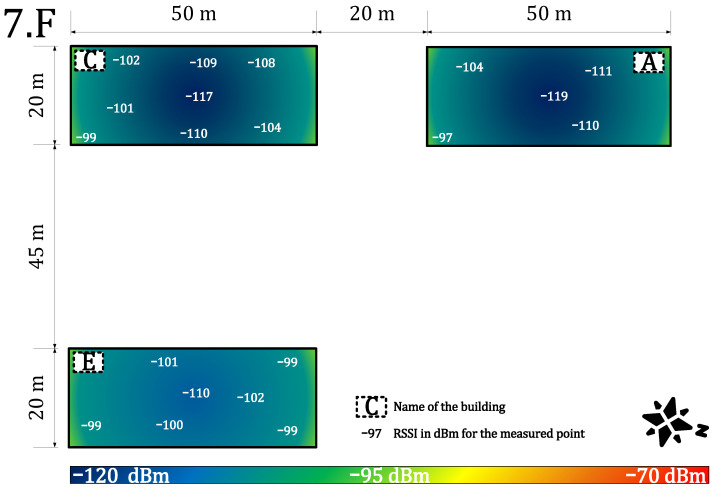
Public network coverage and signal propagation on the seventh floor.

Figure 7 and Figure 8 provide results for both networks on the seventh floor. The public network RSSI ranged from −97 to −119 dBm. The private network RSSI ranged from −70 to −107 dBm with expected best results in the building E (the building with our gateway on the roof). The mean loss rate was <0.1% for both types of network; this allows using both deployments in critical applications requiring >99.9 availability [64]. However, we observed a higher loss rate (7%) under the gateway (approx. 3 m under a gateway with the reinforced concrete roof in between, the building E—the place with highest RSSI −70 dBm, see Figure 7). Although the private network offers higher RSSI than the public network, the public operator offers sufficient service to cover indoor conditions in this case. Based on the authors’ market knowledge, the 868 MHz traffic will probably become denser in the future due to the growing number of sensors. Therefore, we can expect that the selected channels in the frequency plan will gain a higher noise level and the −119 dBm might become a border value for the sensitivity because the interferences might cause a signal strength degradation of over 20 dB for the LoRaWAN technology [62].

**Figure 9:**
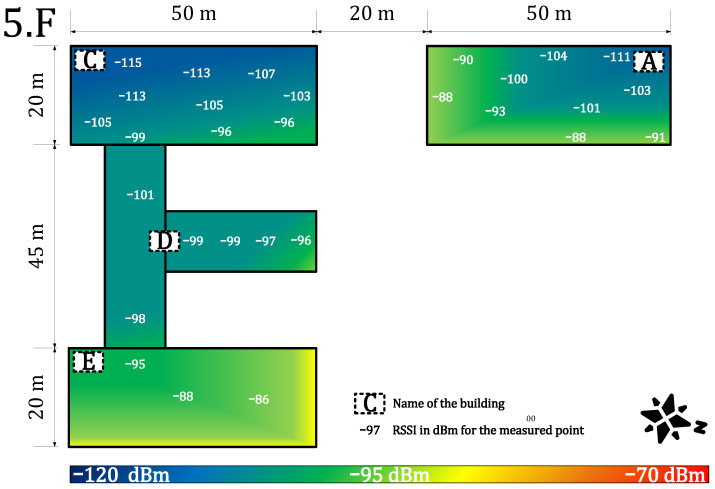
Private network coverage and signal propagation on the fifth floor.

**Figure 10:**
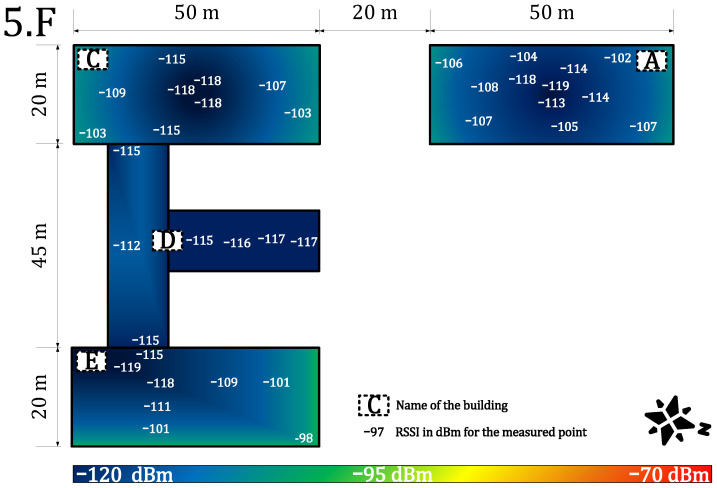
Public network coverage and signal propagation on the fifth floor.

Figure 9 and Figure 10 provide results for both networks on the fifth floor. The private network signal strength was in the range of −86 to −115 dBm with a strength loss of >10 dB in most of the building, except for the building A, where the degradation was minimal due to the line-of-sight between the building and the gateway. The loss rate in the private network grows to 1%. Moreover, the higher loss rate under the gateway in the building E was not observed. On the other hand, the public network signal strength ranged from −98 to −119 dBm. The min/max values are the same as on the previous floor, but the heat-map shows a rapid decrease of the mean RSSI (Figure 10). Further, the average loss rate on the fifth floor grew to 7% in the public network, which allows using it in non-critical indications, metering, and other applications requiring (>90%) availability. On this floor, the private network starts to show a slight advantage.

**Figure 11:**
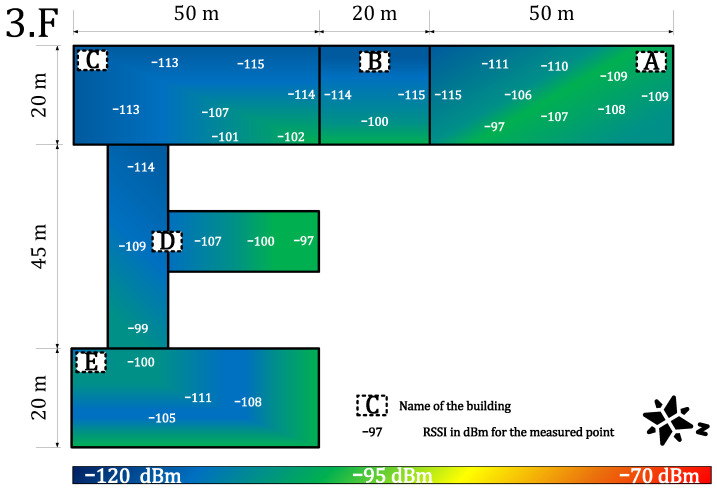
Private network coverage and signal propagation on the third floor.

**Figure 12:**
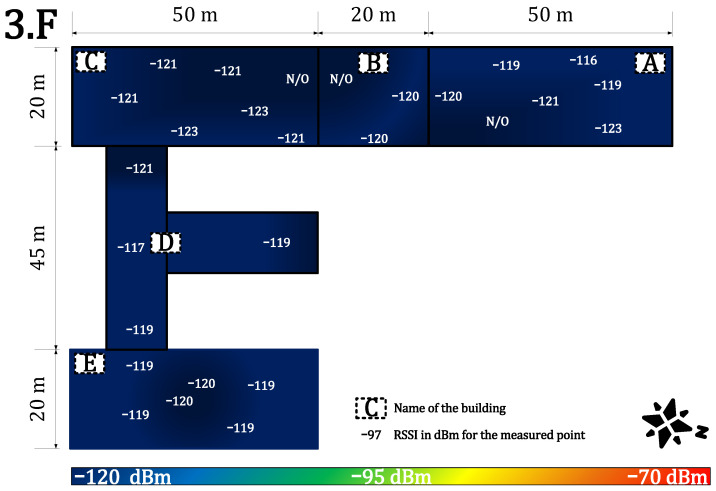
Public network coverage and signal propagation on the third floor.

Figure 11 and Figure 12 provide results for both networks on the third floor. The signal strength for the private network was in the range of −97 to −115 dBm. The signal strength dropped again by about 5 to 10 dB and the loss rate increased to 4%. However, the network parameters were stable, and the communication service was available throughout the whole building. An availability of 96% allows using it in most of the metering applications which require (>90%) availability [64]. Further, the range of RSSI for public networks was in the range of −116 dBm to no signal (N/O). The signal strength also decreased by another 10 dB. Compared with the calibration value, the attenuation is already more than 30 dB, which can be considered as a deep(er) indoor condition. The public service was already unavailable in some parts of the university complex (buildings A, B, and C). In other parts, the service was on the border of the measuring device’s sensitivity. The heat map shows cold signal places throughout the whole building. Further, the average loss rate on this floor increased to 10% for the public network. This is the border value for most of the current applications [64].

**Figure 13:**
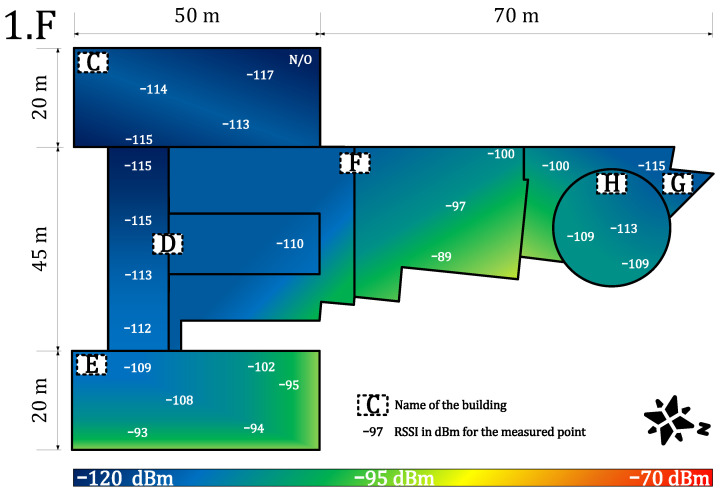
Private network coverage and signal propagation on the first floor.

**Figure 14:**
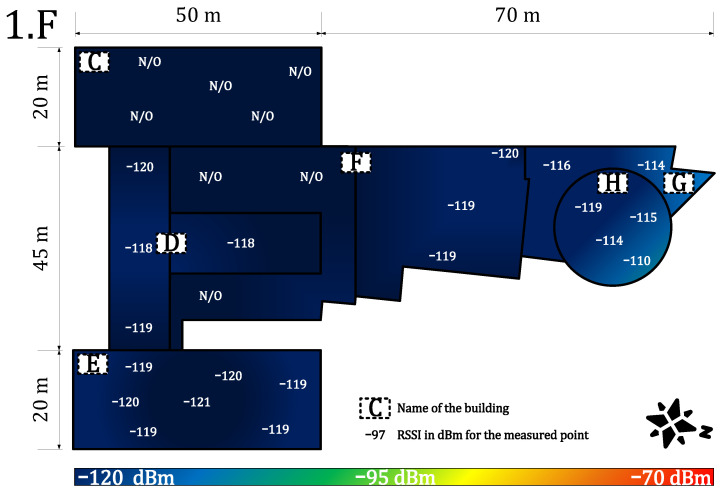
Public network coverage and signal propagation on the first floor.

Figure 13 and Figure 14 provide results for both networks on the first floor. We experienced very deteriorated communication conditions. The building C is in the basement (below ground level). Other floors are above ground. For this reason, the building C shows the worst results. The private network RSSI ranged from −113 to −117 dBm (with no signal in the upper-right corner of the building C—marked as N/O). In the other parts of the campus, the RSSI values ranged from −89 to −115 dBm. The parameters were stable, and the loss rate increased to 5%, which still allows using it in most of the metering applications requiring (>90%) availability [64]. However, the public service was unavailable in the building C, and most of the other parts were on the border of sensitivity, close to −120 dBm. The loss rate grew to 12%, which is unacceptable for most of the current communication applications. The lowest floor, where very deep indoor conditions were experienced, shows the most significant differences between the private and the public network. Both networks show significantly decreased service quality.

The presented results illustratively demonstrate the specifics of the public network’s coverage. The deep middle of the buildings is mostly poorly covered, while the edges feature a higher RSSI benefiting from multiple gateways located around. Meanwhile, our results show that it is possible to cover the whole complex by one single private gateway. For use cases with a higher number of end-nodes, the multiple-gateway solution should be used, i.e., we could add another gateway to the building A or C to improve the network parameters. This shows another advantage of the private network. We can add more gateways to boost the performance where and when needed. Unfortunately, this level of flexibility is hard to achieve when being served by a public network.

### 4.2. Security Evaluation

This section reports the comprehensive security analysis of LoRaWAN and names several benefits of private LoRaWAN deployments. We focus on the basics of security properties and vulnerabilities, and we also mention several improvements in related works. Notably, we analyze how LoRaWAN specification 1.1 (released in October 2017) and the most recent specification 1.0.4(2020) address the security issues present in the previous specification (1.0).

The following LoRaWAN specifications are currently available: 1.0 (2015 [65]), 1.0.1 (2016 [66]), 1.0.2 (2016 [67]), 1.1 (2017 [10]), 1.0.3 (2018 [68]), and 1.0.4 (2020 [69]).

Version 1.0.1 brings many corrections and clarifications to the definitions of the previous specification. Version 1.0.2 separates regional parameters from the link-layer specification. Subsequently, version 1.1 was released, which addresses additional roaming features and security improvements. Consequently, since the industry did not move to version 1.1 and is still building the 1.0 series of infrastructure and products, the LoRa Alliance has created version 1.0.4, which is currently the latest version of the 1.0 series, into which some features from 1.1 are imported. From a security perspective, versions 1.0.1, 1.0.2, and 1.0.3 are almost identical. This is worth noting that since the public networks have been (i) deployed earlier, and (ii) have to host the older sensors, they often base on the older versions of LoRaWAN specification.

The LoRaWAN security design is mainly based on symmetric cryptography. The specifications 1.0 [65] to 1.0.3 [68] define the following main security procedures:

**Key establishment:** The LoRaWAN specification 1.0 offers two approaches to establish keys. The first approach is the OTAA (join procedure). The end-device and the NS (or a JS) generate the AppSKey and NwkSKey keys from the same preshared AppKey. The second approach is the ABP activation. The device address (DevAddr), network session key (NwkSKey), and application session key (AppSKey) parameters are configured at production time. OTAA is considered more secure than ABP, but it still has several security issues.

**Authentication:** Each node has a 64-bit globally unique identifier called device identifier (DevEUI) and a unique 128-bit AES key (called AppKey) that are set by vendors or application providers. The application identifier (AppEUI) uniquely identifies the application. The OTAA proves that both the end-device and the NS (or a JS) have the knowledge of the preshared AppKey key. The end-device sends the join request message with AppEUI, DevEUI, and DevNonce and adds the message integrity code (MIC) computed by AppKey. The NS checks the MIC and generates keys for data encryption and data integrity. The server responds to the end-device by the join accept message with the MIC. The mutual authentication is ensured by the knowledge of the AppKey on both sides.

**Key update:** Session keys can be updated several times, but the preshared master key AppKey cannot be updated.

**Encryption:** Data encryption is ensured by the 128-bit AES encryption in the CTR mode. Application payloads are encrypted by the end-to-end shared key AppSKey, which is known only to the end-device and the AS. Nevertheless, the NS also knows the AppSKey and can decrypt the messages. Therefore, the NS has to be trustworthy.

**Data integrity:** Data integrity is ensured by the 32-bit message integrity code (MIC) produced by the CMAC function using the 128-bit AES encryption. The 4-byte MIC is calculated from a MAC payload and the NwkSKey key shared between the NS and the end-device. This code is added after the MAC payload. To avoid a packet replay attack, a frame counter is used (16 bits). However, the payload could be flipped due to the AES-CTR mode not providing data authentication.

#### 4.2.1. Vulnerabilities

There are several imperfections and security issues of the LoRaWAN technology (specification 1.0.x):

- The preshared key AppKey cannot be updated. The key update problem is discussed in [70].
- The keys are persistently stored on a LoRaWAN device and could be subject to physical attacks. Using a tamper-resistant storage (i.e., secure element, HSM) improves the security of the stored key, but it also increases the costs.
- The paper [71] demonstrates that LoRaWAN transmissions are prone to jamming attacks.
- The paper [72] shows that the OTAA approach enables attackers to conduct a replay attack.
- The operator’s NS knows the application keys and can decrypt the end-to-end communication, as noted in [73] (fixed in 1.0.4).
- The paper [74] shows potential vulnerabilities to denial of service (DoS) attacks during the join procedure.

#### 4.2.2. The State-of-the-Art Improvements

In the following, we analyze the recent works and improvements to the LoRaWAN security approaches.

Kim and Song [70] propose a dual key-based activation scheme. Their proposal resolves the problem of key updates by using a dual key setup. Keys, that users share with the NS and the AS, are recomputed from previous keys and nonces by the AES encryption function. The proposal addresses such security requirements as authentication, message integrity, data confidentiality, and replay attack prevention. End node authentication is achieved by using the shared key with the NS and checking the CMAC value in the join phase.

The public key infrastructure of LoRaWAN has several security issues related to key management and the join phase. For example, the paper [72] demonstrates that attackers may misuse the OTAA for a replay attack. The paper presents this attack and offers the countermeasure by adding a masking token. Kim and Song [73] present a secure D2D link establishment scheme that consists of the SecureD2DReq, and SecureD2DAns messages exchanged between end nodes and the NS. The NS delivers security parameters to both nodes, so that both D2D nodes can securely establish cryptographic keys for protecting the D2D communication. A minor disadvantage is that the NS knows the encryption keys that are used between the nodes. In consequence, the NS has to be a trusted party. The work [75] presents a reputation system in order to select trustworthy nodes as proxies that are involved in the key derivation phase in order to improve the key robustness.

#### 4.2.3. Security Improvements in LoRaWAN™ 1.1 Specification

The specification 1.1 [10] released in 2017 and LoRaWAN™ Backend Interfaces 1.0 Specification [17] enhance the security in several ways and reflect many security issues discovered in the previous version of LoRaWAN standard. The security improvements and differences are as follows:

**Key establishment:** The LoRaWAN specification 1.1 offers a preshared symmetric key approach and OTAA and ABP procedures to derive session keys. However, LoRaWAN 1.1 adds another AES-128 root key, called NwkKey, which is used to derive the FNwkSIntKey, SNwkSIntKey, and NwkSEncKey session keys. This key may be shared with a network operator in order to manage the join procedure and to derive network session keys. The other root key, AppKey, is used for the derivation of the AppSKey session key. The security improvement is that users do not need to share AppKey with the network operator. AppKey and the derived AppSKey can be used solely for end-to-end encryption. Nevertheless, the devices (defined by LoraWAN 1.1) that communicate with NS (defined by LoraWAN 1.0.x) must only use NwkKey to derive all keys in order to preserve backward compatibility. Using the security elements and HSM to store the shared keys is still not possible.

**Authentication:** The version 1.1 improves OTAA (join procedure) by modifying JoinAccept MIC in order to prevent the replay attack. Further, all nonces are not random numbers but counters. Newly, OTAA is managed solely by the JS (not NS), which has to know both shared root keys. The mutual authentication is still based on the secrets shared between the devices and the JS. The knowledge of secrets is proved by computing and checking the CMAC functions (MIC).

**Key update:** Devices supporting LoRaWAN 1.1 can update session keys and reset counters by the rejoin procedure. The size of counters is increased from 16 bits to 32 bits. Nevertheless, the root keys (AppKey, NwkKey) cannot be updated as in 1.0 to 1.0.3.

**Encryption:** Data encryption is ensured by the 128-bit AES encryption in the counter with CBC-MAC (CCM) mode (not only CTR as in 1.0). Newly, the NS is not able to decrypt application data without AppSKey.

**Data integrity:** The version 1.1 defines the CCM authenticated encryption mode that provides data integrity. The data integrity of uplink frames is newly ensured by two CMAC functions with two keys (SNwkSIntKey, FNwkSIntKey). MIC is composed of 2B-cmacS and 2B-cmacF, but the length remains the same (4 B).

A comparison of the security aspects of both the LoRaWAN 1.0 standard and the new LoRaWAN 1.1 standard is displayed in Table 4.

The main improvement of the version 1.1 is in defining another key solely for the network level. This enables an enhancement of security for users and developers in public networks. In this way, users who do not use the operator’s JS can encrypt data at the application layer without being worried about the operator listening. Nevertheless, some security associations between servers are still outside the scope of the LoRaWAN specifications.

#### 4.2.4. Security Improvements in LoRaWAN™ 1.0.4 Specification

Version 1.0.4 brought several improvements from version 1.1. Specifically, the security procedures require the 32-bit frame counter size being stored in persistent memory, such as NVRAM, so that the value remains stored with the rest of the security context during the reboot for the ABP device. As a result, the counter value will not be reset, thus preventing possible threats, and the behavior of DevNonce was changed so that the DevNonce values of the device monotonically increase so that the work of a JS is much easier, and it is possible to monitor DevNonce to prevent replay attacks that are possible when using the same DevNonce.

#### 4.2.5. Security Comparison of Private and Public Network

Due to several security imperfections in the public networks and basic specifications (mainly in 1.0 series), we assume that employing a private network may provide higher security than a public one under certain conditions. The main security benefit of using the private network is that keys are controlled and created by the end-users themselves. There is no possible danger caused by exposing encrypted data to a public operator. The specification 1.1 fixes this issue, but the problem remains if the operator employs the 1.0 series network server. Further, the developers can improve the security in their own private networks by adding security features and procedures presented in the state-of-the-art works, such as device-to-device encryption, reputation approaches, or by solving security associations between servers.

At the same time, the larger public networks have one benefit—they can offer higher availability benefiting from multiconnectivity and presence of multiple gateways, and thus are more resistant to some kinds of attack, such as replay and denial of service attacks. A private network topology with a low number of gateways can be overwhelmed by a large amount of malicious or repeated messages. In these situations, robust public networks could be more stable and reliable.

### 4.3. Deployment Ease Evaluation

This section contains two main parts: (1) costs evaluation, which provides a clear idea of the expected expenses for both public and private networks, and (2) methodology, which discusses the series of steps and operations for deploying the private or public network.

#### 4.3.1. Deployment Costs Evaluation

This section briefly speculates on the costs of the private and the public network. However, an accurate analysis is strongly affected by the business practices and costs in each region and thus is beyond the scope of this paper. We include this analysis to support the evaluation of private and public networks; and to show the main differences in the nature of the costs they inquire. Moreover, this section might also serve for future estimation in specific use cases by application developers.

The costs of technology and solution are based on two types of costs: (i) CAPEX (capital expenditures); and (ii) OPEX (operating expense). The list of items which form the CAPEX costs is displayed in Table 5. Together, these items make up the total CAPEX costs:

(2) $$\begin{array}{c} \sum C_{\mathrm{capex}}=C{}_{\mathrm{prop}}+C{}_{\mathrm{sus}}+C{}_{\mathrm{bld}}+\\ +C{}_{\mathrm{res}}+C{}_{\mathrm{cap}-\mathrm{oth}}-C{}_{\mathrm{dis}}-C{}_{\mathrm{sell}}. \end{array}$$

The CAPEX of a private network highly depends on the size of the network. Based on our experiences, the CAPEX costs of private network are considered to be higher than public network costs due to the high $C{}_{\mathrm{prop}}$ and $C{}_{\mathrm{cap}-\mathrm{oth}}$.

The list of items which form the OPEX is displayed in Table 6.

Together these items result in the total OPEX costs:

(3) $$\begin{array}{c} \sum C_{\mathrm{opex}}=t\;\cdot \;(C{}_{\mathrm{fee}}+C{}_{\mathrm{enr}}+C{}_{\mathrm{rent}1}+\\ +C{}_{\mathrm{rent}2}+C{}_{\mathrm{ope}-\mathrm{oth}}), \end{array}$$

where *t* is the application horizon in years. The OPEX of both networks highly depends on the size of the network. Moreover, the OPEX costs of a private network are considered to be lower than the public network costs, because most of the applications, such as smart grid, smart city, smart home, and others, are considered to be included in the functional user’s infrastructure without any need to build new ones. However, the OPEX costs of the private network will markedly increase if no infrastructure is provided.

The whole costs of the application might be computed as follows:

(4) $$\sum C=C_{\mathrm{capex}}+C_{\mathrm{opex}}.$$

#### 4.3.2. Methodology of Deployment

The public and the private network should follow a certain methodology for deployment. However, our experiences show that these methodologies slightly differ from each other. This chapter introduces a summary of deployment methodology for both private and public networks based on our best practice.

**• Decision- making**

1. Estimate the size of the network (geographic area, number of gateways for a private case, number of nodes, density of nodes), desired service parameters (availability, throughput, type of communication, latency, communication frequency), and desired additional services (such as localization service presence).
2. Determine the possible future growth of the network or the network requirements. In addition, determine the possible future growth of the environment (i.e., new shopping areas, industrial complexes, and other noise-generating elements).
3. Analyze the feasibility of handling these requirements and future needs by LoRaWAN accounting for frequency regulations and technology limitations (check the availability of desired devices for the selected application). Decide whether the LoRaWAN technology is suitable for the selected use case. If possible, make a cost-efficiency analysis for LoRaWAN and other technologies (include the cost of development if end-devices are not available).
4. Use tester device(s) to characterize the signal quality of the public service from the most remote and difficult points (ask for an exact frequency plan of the network service provider). Discuss the possibilities of service-level agreement (SLA) with the public operator. Based on the results, selected parameters, and cost-efficiency, decide whether to go for either the private or the public network.

**• Private Network**

1. Estimation of the network coverage should be computed via simulation tools, i.e., Radio Mobile. This software uses the Irregular Terrain Model (ITS) based on the Longley–Rice model, which is a method for predicting the attenuation of radio signals for a telecommunication link in the frequency range of 20 MHz to 20 GHz. Based on the results, the position of the gateways should be established with reference to the simulation and also the node density. However, the best position may not be always available, and the location of already owned infrastructure should also be considered).
2. Make a noise analysis and select a frequency plan. Deploy the gateways based on the estimated plan.
3. Select power levels and data rates (if adaptive data rate is not in use) for the nodes. Use the tester device to characterize the quality of the signal from the most remote and difficult points.
4. Optimize the network by replacing or adding gateways and device antennas.
5. Deploy the first devices and test the long-term parameters of the network by monitoring the main parameters, i.e., availability and latency.
6. Scale the network up by continuously monitoring the main metrics.
7. The network parameters will change continuously over time, and the network needs to be continuously optimized to preserve the required parameters.

**• Public Network**

1. To ensure the performance metrics, agree with the public operator on the parameters via SLA.
2. Select power levels and data rates (if adaptive data rate is not in use) for the nodes.
3. Deploy the first devices and test the long-term parameters of the network by monitoring the main parameters, i.e., availability or latency.
4. Scale the network up by continuously monitoring the main parameters
5. The network parameters will change continuously over time, and it is necessary to continuously control the SLA from the operator.

### 4.4. Results Discussion

The private approach has a slight advantage over the public approach, because of the possibility of customizing the frequency plan and many other variables. Moreover, our results show a clear advantage of the private network’s performance for an end-user, whose devices are located in a reasonably small geographical area, with respect to coverage (indoor), signal strength, signal propagation, or loss rate. However, the private network will need to increase the number of gateways to provide sufficient communication performance for an increased number of devices or for mobile devices. We provided a methodology for estimating the expenses which impact both of the LoRaWAN approaches. However, these need to be brought into a real-case context. Meanwhile, security-wise, the private approach offers, again, a slight advantage and a higher level of security, mostly thanks to the private key management without a third party and the possibility of improving the internal security mechanisms.

## 5. Conclusions and Future Work

This article clarifies the differences between the private and the public deployments of the LoRaWAN technology by providing theoretical and experimental results. We expect that both types of LoRaWAN deployments will need to face the inevitable issues of growing of the background noise level caused by the increasing number of devices in the unlicensed band. The results presented in this study demonstrate the importance of frequency resource usage optimization.

The number of the gateways and their optimization in the context of both private and public network is another issue of the utmost importance, requiring further research. Notably, in the current study we focused on the uplink-only traffic, which is specific for sensor devices. Meanwhile, the downlink traffic (relevant, e.g., for actuator devices) introduces a number of novel challenges and optimization dimensions, which can also affect the interplay between public and private deployments. One of the notable issues here is the half-duplex nature of many commercial gateways and uplink–downlink interference.

In addition, in the paper, we approached and discussed the security aspects and cost structures for both private and public networks. We identified the different trade-offs between the parameters and performance metrics, and showed that under particular implications either of the approaches may outperform its counterpart. This justifies the need for further research to enable development of more accurate and easy-to-use models, which can be used to plan and assess the deployment of LoRaWAN networks. This is the challenge we aim to approach in our further studies.

## Figures and Tables

**Table 1:** Comparison of different parameter estimations based on the physical level in selected papers.

Paper	Data Rate (kb/s)	Communication Range (km)
[25]	0.29–50	15
[13]	0.29–50	less than 35
[5]	max. 50	5 (U), 20 (R)
[6]	0.3–37.5	3–10 (U), 30–50 (R)
[26]	0.3–37.5 (L), 50 (F)	10 (U), 50 (R)
[27]	27 (L), 50 (F)	2–5 (U), 15 (R)
[7]	0.25/5.5/11/50	2–15
[9]	max.50	5 (U), 20 (R)
[28]	0.3–50	up to 10
[29]	0.3–50	2–5 (U)
[30]	0.29–50	2–5 (U) 45 (R)

Note: L —LoRa; F—FSK; U—Urban; R—Rural.

**Table 2:** Frequency plan for experimental measurements (both public and private networks).

Channels, f [MHz]	868.1 *	868.3 *	868.5 *	867.1
Channels (cont-d), f (MHz)	867.3	867.5	867.7	867.9
BW (KHz)	125			
MOD/SF	LoRa with Multi-SF			

* Default LoRaWAN channels from the newest specification [10].

**Table 3:** Effect of the plan on performance (arithmetic means).

Scenario	RSSI (dBm)	SNR (dB)	LR (%)
Public—default plan (i)	−125	−18.36	43
Public—default plan (ii)	−97	−2.05	3
Private—default plan (i)	−96	0.01	21
Private—extended plan (ii)	−70	14.90	1

**Table 4:** Differences in security parameters for LoRaWAN 1.0.x and LoRaWAN 1.1.x.

Security Procedure	LoRaWAN 1.0.x	LoRaWAN 1.1.x
Key establishment	OTAA/ABP	Added the second root key and enhanced key derivation
Authentication	64 b/128 b keys	Improved anti-replay technique
Key update	Only session key	Session key enhanced by the rejoin procedure
Encryption	AES${}_{\mathrm{CTR}}$-128	AES${}_{\mathrm{CCM}}$-128 and enhanced data confidentiality at the application layer
Data integrity	32 b MIC	Provided by AES-CCM (2CMAC functions)

Data obtained from the standard specification [9,10,11,12,13,14,15,16,17,18,19,20,21,22,23,24,25,26,27,28,29,30,31,32,33,34,35,36,37,38,39,40,41,42,43,44,45,46,47,48,49,50,51,52,53,54,55,56,57,58,65,67].

**Table 5:** The list of components of CAPEX costs.

Parameter	Description
$C{}_{\mathrm{prop}}$	From the end-user perspective, in the case of public network, the propriety costs are mainly composed of the costs of the end-devices. However, the private networks must also include other costs, i.e., gateways, racks, cables, antennas, feeders, software customized solutions, and others. Moreover, the costs for network optimization must include covering the places with a higher noise level (for an estimation of a precise simulation model needs to be made). If a larger private network is considered, it might also be necessary to include core infrastructure building costs if needed.
$C{}_{\mathrm{sus}}$	Sustainability needs to be included if the horizon of the considered application is beyond the device’s lifetime (i.e., devices with a 15-year lifetime will be used in applications with a horizon of 30 years).
$C{}_{\mathrm{bld}}$	If a large-scale private network is considered, there might also be additive costs for renting roofs or buildings for gateways. However, this item considers only the buying price for the buildings, where the fees (if any) are included in the similar item for the OPEX costs.
$C{}_{\mathrm{res}}$	The LoRaWAN technology is still quite new on the market, and most of the end-devices are of basic character. More specific applications could require research of the end-devices, which will also impact the final CAPEX costs.
$C{}_{\mathrm{cap}-\mathrm{oth}}$	There might be other additional costs not mentioned above, i.e., high-level design (HLD), low-level design (LLD), detailed-level design (DLD), installation and deployment costs, device configuration, supplies or network optimization costs (work).
$C{}_{\mathrm{dis}}$	There will be a certain discount on the price of the devices (item $C_{\mathrm{prop}}$) based on the seller and amount of devices.
$C{}_{\mathrm{sell}}$	If the time difference between the application horizon and the device lifetime is >0, there is a possibility of selling the network equipment, which slightly lowers the CAPEX costs (i.e., the devices with a 15-year lifetime will be used in an application with a horizon of 5 years).

**Table 6:** The list of items constituting OPEX.

Parameter	Description
$C{}_{\mathrm{fee}}$	In the public network, there will be regular fees based on the number of devices and the number of messages. Though the private network has no device or message fees, the fee for the back-end (LoRaWAN server) needs to be considered. Moreover, the gateways need to be connected via a transport technology such as cellular or Ethernet. For this reason, there might be additional costs for transport services (connecting the gateways). Further, this item also includes regular fees such as software licenses, software updates, and paid support.
$C{}_{\mathrm{enr}}$	The price for energy consumption. The public network mostly contains only end-devices which are often powered by batteries (devices powered by power network should be included here). However, the private network’s energy consumption costs must include the cooling system energy costs as well as main servers, gateways, and other devices used for the LoRaWAN infrastructure. Moreover, this part should also include battery exchange, which occurs in the use cases with a higher frequency of messages, where batteries last only several years.
$C{}_{\mathrm{rent}1}$	These are the rents for roofs or pillars for antennas, which are necessary in the private networks (large-scale private applications only).
$C{}_{\mathrm{rent}2}$	Second renting part, where rented parts of the infrastructure might be included (again, mostly in the large-scale private applications only).
$C{}_{\mathrm{ope}-\mathrm{oth}}$	Other operational costs such as material costs, insurance, surveillance, training, taxes, salaries, depreciation, and others.

Note: All the costs must be computed for one whole year.

## Data Availability

All data are available on demand via corresponding author.
